# Comparison of Prevalence- and Smoking Impact Ratio-Based Methods of Estimating Smoking-Attributable Fractions of Deaths

**DOI:** 10.2188/jea.JE20150058

**Published:** 2016-03-05

**Authors:** Kyoung Ae Kong, Kyung-Hee Jung-Choi, Dohee Lim, Hye Ah Lee, Won Kyung Lee, Sun Jung Baik, Su Hyun Park, Hyesook Park

**Affiliations:** 1 Clinical Trial Center, Ewha Womans University Medical Center, Seoul, Korea; 2 Department of Preventive Medicine, School of Medicine, Ewha Womans University, Seoul, Korea; 3 Department of Social and Preventive Medicine, Inha University School of Medicine, Incheon, Korea

**Keywords:** smoking, population-attributable fraction, risk assessment, population health

## Abstract

**Background:**

Smoking is a major modifiable risk factor for premature mortality. Estimating the smoking-attributable burden is important for public health policy. Typically, prevalence- or smoking impact ratio (SIR)-based methods are used to derive estimates, but there is controversy over which method is more appropriate for country-specific estimates. We compared smoking-attributable fractions (SAFs) of deaths estimated by these two methods.

**Methods:**

To estimate SAFs in 2012, we used several different prevalence-based approaches using no lag and 10- and 20-year lags. For the SIR-based method, we obtained lung cancer mortality rates from the Korean Cancer Prevention Study (KCPS) and from the United States-based Cancer Prevention Study-II (CPS-II). The relative risks for the diseases associated with smoking were also obtained from these cohort studies.

**Results:**

For males, SAFs obtained using KCPS-derived SIRs were similar to those obtained using prevalence-based methods. For females, SAFs obtained using KCPS-derived SIRs were markedly greater than all prevalence-based SAFs. Differences in prevalence-based SAFs by time-lag period were minimal among males, but SAFs obtained using longer-lagged prevalence periods were significantly larger among females. SAFs obtained using CPS-II-based SIRs were lower than KCPS-based SAFs by >15 percentage points for most diseases, with the exceptions of lung cancer and chronic obstructive pulmonary disease.

**Conclusions:**

SAFs obtained using prevalence- and SIR-based methods were similar for males. However, neither prevalence-based nor SIR-based methods resulted in precise SAFs among females. The characteristics of the study population should be carefully considered when choosing a method to estimate SAF.

## INTRODUCTION

Estimating disease burden and quantifying the relative contributions of various risk factors can inform prevention strategies at a national level. Country-specific risk assessments of disease burden have been undertaken since the 1990 Global Burden of Disease (GBD) study, the first global comparative risk assessment.^1^

Smoking is a leading cause of cancers, heart disease, and chronic lung disease.^2^ According to the GBD study, 6.3 million deaths worldwide were attributable to smoking and secondhand smoking in 2010, rendering it the second leading contributor to disease burden.^1^ Because it is a major modifiable risk factor for premature mortality, estimating the burden attributable to smoking is particularly important, and several methods have been developed to measure it.^3^^–^^6^ The most common is the prevalence-based method, followed by the smoking impact ratio (SIR) method.^4^

The prevalence-based method is calculated using the prevalences of current and former smoking. However, national prevalence data are frequently unavailable, and more importantly, the current prevalence of smoking may not be a good indicator of smoking hazards accumulated in previous years because it cannot integrate smoking history information pertaining to age at initiation, duration and intensity of smoking, quitting, type of cigarette smoked, and particular patterns of smoking behavior.^5^^,^^7^ The incongruity of using current smoking prevalence to calculate the deaths attributable to smoking exposure in previous years has led to the use of a time-lagged prevalence approach.^5^

The SIR, an indirect indicator of the cumulative exposure to smoking in a given population, is defined as excess population lung cancer mortality for never-smokers relative to excess lung cancer mortality for a known reference group of smokers, adjusted to account for differences in never-smoker lung cancer mortality rates across the population.^1^^,^^5^^–^^9^ SIR presents the accumulated smoking hazards of the study population as a proportion of smokers among a reference population, where relative risks (RRs) for other diseases are known and lung cancer mortality rates of smokers and never-smokers are the same as those of the study population.^7^^–^^11^

In the Republic of Korea, there has been a rapid decrease in smoking prevalence, and all data sources necessary for calculating the smoking-attributable fraction (SAF) of deaths are available: a national dataset for smoking prevalence, relative risks for various diseases determined from a large-scale cohort, and the lung cancer mortality rates of smokers and never-smokers. For risk assessment of disease burden to inform national prevention strategies, a comparison of SIR- and prevalence-based methods may be useful. Although several studies have indicated that estimates using the two methods are similar (although the SIR-based estimates tend to be higher, particularly for cardiovascular diseases [CVDs] and diabetes and among females),^3^^,^^6^ no such study has been conducted in Asian countries.

In this study, we compared the SAF of deaths in Korea using prevalence- and SIR-based methods with several different approaches based on the reference population and lag time.

## METHODS

### Smoking exposure measures

#### Prevalence-based method

Four approaches with three prevalence datasets were employed: 1) prevalence of current (CS) and former smoking (FS) with no lag period (no lag-CS+FS prevalence); 2) 10-year-lagged prevalence of only CS (lag10yr-CS prevalence); 3) 10-year-lagged prevalence of both CS and FS (lag10yr-CS+FS prevalence); and 4) 20-year-lagged prevalence of only CS (lag20yr-CS prevalence). Current and 10-year-lagged prevalence data according to sex and 5-year age group were extracted from the 2012 and 2001 Korea National Health and Nutrition Examination Surveys,^12^ and 20-year-lagged prevalence data according to sex and 10-year age group were taken from the 1990 Survey on the Smoking Habits in Korea.^13^

#### SIR-based method

We estimated three types of SIR using the Korean Cancer Prevention Study (KCPS) and Cancer Prevention Study-II (CPS-II): 1) SIR based on the lung cancer mortality rates of smokers and never-smokers derived from the KCPS (KCPS-SIR); 2) SIR based on estimated lung cancer mortality rates among older-aged KCPS smokers (adjusted KCPS-SIR); and 3) SIR based on the lung cancer mortality rates of smokers and never-smokers derived from the CPS-II (CPS-II-SIR).

The KCPS included a cohort of 1.3 million males and females 30–95 years of age who participated in a National Health Insurance Corporation medical evaluation between 1992 and 1995.^14^^–^^17^ The majority of males in the cohort were ever-smokers (current, 59%; former, 21%); 30% of the current smokers smoked ≥20 cigarettes per day. Lung cancer mortality rates were obtained from a comprehensive study of lung cancer^18^ in which KCPS never-smokers were followed-up to 2004, with the follow-up of current smokers limited to 1998 to allow for comparison with the CPS-II and to minimize the effects of cessation. Additionally, we compared KCPS lung cancer mortality rates with those of a pooled analysis of three large-scale cohort studies in Japan (Japan Public Health Center-Based Prospective Study, Japan Collaborative Cohort Study, and Three-Prefecture Cohort Study [3-pref study]) (J3CS) among males and those of the 3-pref study among females.^18^^–^^20^ We then estimated lung cancer mortality rates in KCPS smokers aged ≥75 years using a log-linear regression model to calculate the adjusted KCPS-SIR because they were markedly lower than those of the J3CS/3-pref study and those in the Korean population in 2012, rendering the original KCPS-SIR larger than 1.

The CPS-II was a prospective cohort study conducted by the American Cancer Society. Approximately 1.2 million males and females were enrolled in 1982 and followed-up to 1988.^8^^,^^9^^,^^21^ The majority of males were current, lifelong smokers with a mean consumption of approximately 20 cigarettes per day. We used the CPS-II data to estimate SIR in accordance with the majority of previous GBD studies. The CPS-II study was conducted when the effects of smoking on mortality fully appeared; the study also included RRs for diseases associated with smoking, adjusted for important covariates.^6^^,^^10^^,^^22^ CPS-II lung cancer mortality rates were retrieved from the National Cancer Institute Monograph 8.^21^

The background SIR was calculated using the following formula:

$$\text{SIR}=[({\text{C}}_{\text{LC}}-{\text{N}}_{\text{LC}})/({\text{S}}^{*}{}_{\text{LC}}-{\text{N}}^{*}{}_{\text{LC}})]\times ({\text{N}}^{*}{}_{\text{LC}}/{\text{N}}_{\text{LC}}),$$

where C_LC_ and N_LC_ are the lung cancer mortality rates of the overall study population and of the never-smokers therein, respectively; S^*^_LC_ and N^*^_LC_ represent the lung cancer mortality rates of smokers and never-smokers in a reference population, respectively.^5^^,^^7^^–^^9^ The present study used the lung cancer mortality rates of KCPS never-smokers as N_LC_.

### Relative risks for diseases associated with smoking

The RRs of diseases associated with smoking used to estimate SAFs are listed in Table 1. We used the relative risks for diseases from KCPS in estimating prevalence- and KCPS-SIR-based SAFs.^14^^,^^15^^,^^17^^,^^23^^,^^24^ For CVDs and chronic obstructive pulmonary disease (COPD), age-specific RRs were not available from the KCPS; therefore, we used RRs from the J3CS,^25^^–^^27^ as the sample populations are comparable. To estimate CPS-II-SIR-based SAFs, RRs from the CPS-II were used.^6^^,^^8^^,^^9^^,^^22^^,^^28^ RRs were adjusted for important covariates for each disease; RRs for cancers in former smokers and for tuberculosis in current and former smokers in KCPS were age adjusted; RRs for CVDs in J3CS were adjusted for age and cohort.

**Table 1.** Relative risks for diseases associated with tobacco smoking

Disease (ICD-10 code)	Age	Males				Females			
	
		KCPS^14^^,^^15^^,^^17^^,^^23^^–^^27^		CPS-II^6^^,^^8^^,^^9^^,^^22^^,^^28^		KCPS^14^^,^^15^^,^^17^^,^^23^^–^^27^		CPS-II^6^^,^^8^^,^^9^^,^^22^^,^^28^	
			
		Current	Former	Current	Former	Current	Former	Current	Former
Mouth and oropharyngeal cancer (C00–C14)		2.18	1.89^c^	8.10	4.40	1.97^c^	1.23^c^	6.00	3.00
Esophageal cancer (C15)		3.60	1.90	8.10	4.40	3.60^d^	1.90^d^	6.00	3.00
Laryngeal cancer (C32)		6.50	3.60	8.10	4.40	4.21		6.00	3.00
Lung cancer (C33–C34)		4.60	2.20	21.30	8.30	2.83	1.70	12.50	4.80
Stomach cancer (C16)		1.60	1.40	2.16	1.55	1.10	1.00	1.49	1.36
Liver cancer (C22)		1.40	1.30	2.33	1.81	1.13	1.30	1.50	1.69
Pancreatic cancer (C25)		1.50	1.30	2.20	1.20	1.21	0.80	2.20	1.60
Colorectal cancer (C18–C20)		1.11	1.10	1.32	1.15	1.16	1.10^d`^	1.41	1.22
Kidney cancer^a^ (C64)		1.29	1.20	2.50	1.80	1.63	1.20^d^	1.50	1.20
Bladder cancer^b^ (C65–C68)		2.25	1.60	3.00	2.00	1.65	1.60^d^	2.40	2.00
Cervical and uterine cancer (C53)						1.91	1.90	1.50	1.40
Leukemia (C91–C95)		1.30	1.40	1.89	1.30	1.10	0.96^c^	1.23	1.30
Total CVD (I10–I99)	40–64	1.77^c^	1.26^c^			2.68^c^	1.74^c^		
	≥65	1.33^c^	1.13^c^			1.50^c^	1.39^c^		
Ischemic heart disease (I20–I25)	40–44	4.08^c^	1.78^c^	5.51	1.18	2.47^c^	2.79^c^	2.26	2.22
	45–59	2.50^c^	1.78^c^	3.04	1.64	4.36^c^	2.79^c^	3.78	1.74
	60–64	2.19^c^	1.78^c^	1.88	1.29	3.10^c^	2.79^c^	2.53	1.34
	65–69	2.19^c^	1.68^c^	1.88	1.29	3.10^c^	2.22^c^	2.53	1.34
	70–79	1.92^c^	1.68^c^	1.44	1.13	2.21^c^	2.22^c^	1.68	1.40
	≥80	1.09^c^		1.05	1.02	1.64^c^		1.38	1.40
Stroke (I60–I69)	40–44	1.41^c^	0.97^c^	3.12^e^	0.84^e^	2.75^c^	1.85^c^	4.61^e^	1.44^e^
	45–59	1.41^c^	0.97^c^	3.12	0.84	2.75^c^	1.85^c^	4.61	1.44
	60–64	1.26^c^	0.97^c^	1.87	1.19	1.85^c^	1.85^c^	2.81	1.44
	65–69	1.26^c^	1.02^c^	1.87	1.19	1.85^c^	1.09^c^	2.81	1.44
	70–79	1.13^c^	1.02^c^	1.39	1.00	1.24^c^	1.09^c^	1.95	1.36
	≥80	1.02^c^		1.05	0.78	0.98^c^		0.94	0.93
Hypertensive disease (I10–I13)				1.96	1.00			2.12	1.12
Other CVDs (I00–I09, I26–I51, I70–I99)				2.15	1.30			2.00	1.34
Diabetes (E10–E14)		1.44	0.96	1.42	1.10	1.86	1.33	1.14	0.89
COPD (J40–J44)		3.09^c^	2.76^c^	10.80	7.80	3.55^c^	1.16^c^	12.3	8.90
Asthma (J45–J46)		3.60	3.10			3.60^d^	3.10^d^		
Lower RTI (J12–J18)		1.17^c^	1.09^c^			1.39^c^	1.40^c^		
Pulmonary TB (A15–A16)		1.21	1.37	1.62^f^	1.58^f^	1.08	1.98	1.62^f^	1.58^f^
Other respiratory diseases (J09–18, J45–J46)				1.90	1.40			2.20	1.20

COPD, chronic obstructive pulmonary disease; CPS-II, Cancer Prevention Study-II of the American Cancer Society; CVD, cardiovascular disease; ICD-10, International Classification of Diseases - 10th Revision; KCPS, Korean Cancer Prevention Study; RR, relative risk; RTI, respiratory tract infection; TB, tuberculosis.

^a^CPS-II: kidney and other urinary cancers (C64–C65, C68).

^b^CPS-II: bladder cancer (C67).

^c^RR of the three large-scale cohort studies in Japan (J3CS).

^d^RR of males.

^e^RRs of participants 45–59 years of age.

^f^RRs derived from a meta-analysis.

### Calculation of the smoking-attributable fraction of mortality

SAFs were calculated using an attributable-fraction formula applicable to multiple categories.^29^^–^^31^ Using the prevalence-based method, the SAF for each disease according to sex and age group was calculated using the following formula:

$$\begin{array}{l} \text{SAF}(\%)=100\times [{\text{P}}_{\text{FS}}\times ({\text{RR}}_{\text{FS}}-1)\\ \;+{\text{P}}_{\text{CS}}\times ({\text{RR}}_{\text{CS}}-1)]/[{\text{P}}_{\text{FS}}\times ({\text{RR}}_{\text{FS}}-1)\\ \;+{\text{P}}_{\text{CS}}\times ({\text{RR}}_{\text{CS}}-1)+1], \end{array}$$

P_FS_ and P_CS_ represent the prevalence of former and current smokers, respectively, and RR_FS_ and RR_CS_ represent the RRs of former and current smokers, respectively.

For the SIR-based method, the SAF was calculated using the following formula:

$$\begin{array}{l} \text{SAF}(\%)=100\times \text{SIR}\\ \;\times ({\text{RR}}_{\text{CS}}-1)/[\text{SIR}\times ({\text{RR}}_{\text{CS}}-1)+1]. \end{array}$$

The overall SAF for each disease was calculated as a weighted average of age-specific SAFs according to the number of deaths in each age group. The number of cause-specific deaths and the lung cancer mortality rates according to sex and 5-year age group in 2012 were obtained from Statistics Korea.^32^ The analysis was restricted to the population ≥40 years of age and was conducted separately by sex. This study analyzed publicly available datasets and was therefore exempt from institutional review board approval.

## RESULTS

### Relative risks and prevalence of smoking and smoking impact ratio

For current smokers, the RRs for laryngeal, esophageal, and lung cancer, ischemic heart disease (IHD) among age groups <60 years, COPD, and asthma were relatively high (>2.5) in the KCPS (Table 1). RRs were higher in the CPS-II study, particularly for lung cancer and COPD, although J3CS and CPS-II RRs for IHD were similar. For the majority of the diseases, RRs for former smokers were much lower than for current smokers.

Among males, current smoker prevalence declined steeply, and former smoker prevalence increased with age (Table 2). Current smoker prevalence in 2012 was lower than that in 2001 and 1990 in all age groups, by approximately 15 and 30 percentage points, respectively. Ever-smoker prevalence rates across 10-year birth cohorts were stable. For example, the current smoker prevalence of males aged 40–49 years in 1990 was 72.9%, and the ever-smoker prevalence was approximately 78% in males aged 50–59 years in 2001 and 60–69 years in 2012. In females, current smoker prevalence was <7% for the majority of the age groups, but was markedly higher in females ≥60 years of age (29.5% in 1990 and 11% in 2001), although this decreased to 2.4% in 2012.

**Table 2.** Smoking impact ratios and prevalence of current and former smoking in Korea

Age	Prevalence of smoking					Smoking impact ratio		
	
	2012		2001		1990		2012	
			
	Current	Former	Current	Former	Current	KCPS	KCPS, adjusted^a^	CPS-II
Males								
20–24					0.777			
25–29					0.777			
30–34			0.654	0.135	0.791			
35–39			0.705	0.133	0.791			
40–44	0.525	0.262	0.699	0.126	0.729	0.488	0.488	0.344
45–49	0.465	0.388	0.628	0.204	0.729	0.458	0.458	0.086
50–54	0.436	0.396	0.575	0.211	0.732	0.375	0.375	0.051
55–59	0.394	0.457	0.538	0.232	0.732	0.611	0.611	0.046
≥60	0.254	0.527	0.445	0.353	0.689			
60–64	0.267	0.485	0.490	0.273		0.544	0.544	0.091
65–69	0.273	0.550	0.510	0.327		0.679	0.679	0.099
≥70	0.232	0.548	0.338	0.464				
70–74	0.208	0.574				0.696	0.696	0.110
75–79	0.308	0.459				1	0.790	0.150
≥80	0.117	0.688				1	0.546	0.286

Females								
20–24					0.015			
25–29					0.015			
30–34			0.042	0.012	0.014			
35–39			0.029	0.009	0.014			
40–44	0.060	0.039	0.030	0.007	0.033	0	0	0
45–49	0.049	0.015	0.047	0.001	0.033	0	0	0.005
50–54	0.096	0.019	0.051	0.010	0.113	0.075	0.075	0.015
55–59	0.058	0.034	0.028	0.011	0.113	0.178	0.178	0.022
≥60	0.024	0.035	0.110	0.028	0.295			
60–64	0.018	0.014	0.057	0.020		0.207	0.207	0.016
65–69	0.014	0.006	0.068	0.011		0.171	0.171	0.025
≥70	0.032	0.059	0.180	0.044				
70–74	0.037	0.032				0.313	0.313	0.080
75–79	0.038	0.054				0.688	0.245	0.077
≥80	0.009	0.122				1	0.300	0.210

CPS-II, Cancer Prevention Study-II; KCPS, Korean Cancer Prevention Study.

^a^Smoking impact ratio of the KCPS, adjusted for participants 75–79 and ≥80 years of age based on the estimated lung cancer mortality rates of KCPS smokers according to a log-binomial regression analysis.

Lung cancer mortality rates among never-smokers in the KCPS and J3CS/3-pref study were higher than those in CPS-II (Figure A–B). The rates among CPS-II smokers were markedly higher than other Asian cohorts. Lung cancer mortality rates increased with age in all cohorts and in the general Korean population in 2012. However, lung cancer mortality rates among KCPS smokers abruptly decreased in older age groups and were significantly lower than the J3PS/3-pref study and the general Korean population in 2012. Accordingly, the KCPS-SIR, which was approximately 0.5–0.7 for males and 0.1–0.2 for females in the majority of age groups, abruptly increased to 1 in males ≥75 and in females ≥80 years of age (Table 2). After adjusting lung cancer mortality in older age groups, the KCPS-SIRs were similar to those in younger age groups. The CPS-II-SIRs were <0.1 for the majority of the age groups, which were markedly lower than the KCPS-SIRs.

**Figure.**
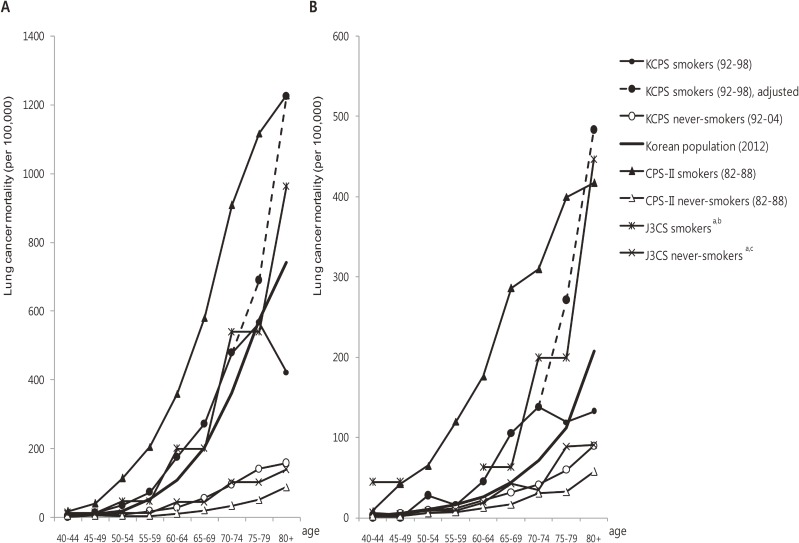
Lung cancer mortality rates of smokers and non-smokers in the Korean Cancer Prevention Study (KCPS), Cancer Prevention Study-II (CPS-II), three large-scale cohort studies in Japan (J3CS), and the general Korean population. A, lung cancer mortality rates among males; B, lung cancer mortality rates among females, from the KCPS, CPS-II, and J3CS^a,b,c^ and the general Korean population in 2012. ^a^Lung cancer mortality rates among men in the pooled analysis of the three large-scale cohort studies in Japan.^20^ ^b^Smokers’ lung cancer mortality rates among women are depicted with values approximated from the graph of the three-prefecture cohort study.^19^ ^c^Never-smokers’ lung cancer mortality rates among women in the three-prefecture cohort study.^18^

### Smoking-attributable fractions of deaths

Among males, differences in SAFs by time-lagged prevalence period were <10 percentage points for the majority of diseases (Table 3). The highest SAFs for the majority of diseases were derived using lag20yr-CS prevalence, followed by lag10yr- and no lag-CS+FS, and then by lag10yr-CS prevalences. The lag10yr-CS+FS prevalence-based SAFs were 39.4% for cancers, 20.9% for total CVD, and 32.4% for respiratory diseases.

**Table 3.** Estimated smoking-attributable fraction of deaths among Korean males in 2012 calculated using smoking impact ratio- and prevalence-based methods

Disease	Number of deaths in 2012	Prevalence-based SAF (%)				SIR-based SAF (%)		
	
		*No lag* *(2012)*	*10-year lag* *(2001)*		*20-year lag* *(1990)*	KCPS	KCPS, adjusted^a^	CPS-II
		
		Current +Former	Current	Current +Former	Current			
Cancers	38 630	35.6	34.7	39.4	41.1	40.4	37.9	32.2
Mouth, pharyngeal	791	44.4	39.0	46.6	46.7	43.9	41.6	42.6
Esophageal	1276	53.7	57.1	61.4	65.6	64.7	62.1	44.8
Laryngeal	388	73.5	72.7	77.8	80.0	80.2	77.6	47.5
Lung	12 106	60.8	63.9	68.3	72.4	72.6	69.5	70.6
Stomach	5980	27.2	23.7	29.8	30.6	30.0	26.8	13.2
Liver	8333	21.5	18.4	22.9	23.0	20.4	18.9	12.6
Pancreatic	2593	22.8	20.8	25.7	26.9	26.1	23.7	12.8
Colorectal	4630	7.6	5.3	7.8	7.5	7.6	6.4	4.3
Kidney^b^	656	15.7	13.4	17.2	17.7	16.9	15.0	16.0
Bladder^c^	1126	38.4	36.6	43.9	47.4	50.4	44.1	24.8
Leukemia	751	22.5	13.9	21.0	18.2	17.0	15.2	10.7
Total CVDs^d^	27 126	16.7	17.8	20.9	23.7	24.0	20.3	8.8
IHD	7553	30.8	29.6	33.9	35.3	33.8	31.8	7.1
Stroke	12 150	5.2	7.8	8.1	10.0	9.2	8.4	5.8
Diabetes	5783	10.1	18.0	18.0	24.3	25.0	21.5	5.8
Respiratory diseases^e^	11 350	31.3	24.2	32.4	31.9	35.1	29.7	31.4
COPD	4016	59.4	46.9	60.8	59.8	64.7	56.6	63.2
Asthma	723	63.9	52.3	65.5	65.0	69.1	61.4	.
Lower RTI	5161	8.1	6.8	9.7	10.8	13.1	9.5	.
Pulmonary TB	1450	20.2	9.3	17.9	13.3	14.3	11.4	9.2

COPD, Chronic obstructive pulmonary disease; CPS-II, Cancer Prevention Study-II; CVD, cardiovascular disease; IHD, ischemic heart disease; KCPS, Korean Cancer Prevention Study; RTI, respiratory tract infection; SIR, smoking impact ratio; SAF, smoking attributable fraction of deaths; TB, tuberculosis.

^a^KCPS, adjusted: SAF estimated using SIR for participants 75–79 and ≥80 years of age based on the estimated lung cancer mortality rates of KCPS smokers according to a log-binomial regression analysis.

^b^CPS-II: kidney and other urinary tract cancers (number of deaths in 2012 = 865).

^c^CPS-II: bladder cancer (number of deaths in 2012 = 917).

^d^IHD: stroke, hypertensive disease, and other CVDs (I10–I99).

^e^COPD: asthma, lower RTI, and pulmonary TB.

The KCPS-SIR-based SAFs were as follows: 40.4% for cancers, 24.0% for total CVD, and 35.1% for respiratory diseases. Adjusted KCPS-SIR-based SAFs were estimated to be lower by approximately 3 percentage points. CPS-II-SIR-based SAFs were markedly lower compared with those of the KCPS for most diseases, except lung cancer and COPD. There were differences of >15 percentage points between the KCPS and CPS-II SAFs for several diseases, including IHD and laryngeal cancer.

Prevalence-based and SIR-based methods using a national cohort produced similar estimates for males. Adjusted KCPS-SIR- and lag10yr-CS+FS prevalence-based SAFs, as well as original KCPS-SIR- and lag20yr-CS prevalence-based SAFs, differed by <5 percentage points for the majority of diseases.

Among females, differences among SAFs according to time-lagged prevalence period were <10 percentage points for the majority of diseases, but the largest difference was 32 percentage points for COPD (Table 4). Lag20yr-CS prevalence-based SAFs were the highest for the majority of diseases, followed by lag10yr-CS+FS and lag10yr-CS prevalences (which were highly similar to each other) and no lag-CS+FS prevalence-based SAFs. Lag10yr-CS+FS prevalence-based SAFs were 5.5% for cancers, 7.5% for total CVDs, and 13.6% for respiratory diseases.

**Table 4.** Estimated smoking-attributable fraction of deaths among Korean females in 2012 calculated using smoking impact ratio- and prevalence-based methods

Disease	Number of deaths in 2012	Prevalence-based SAF (%)				SIR-based SAF (%)		
	
		*No lag* *(2012)*	*10-year lag* *(2001)*		*20-year lag* *(1990)*	KCPS	KCPS, adjusted^a^	CPS-II
		
		Current + former	Current	Current + former	Current			
Cancer	18 457	3.4	5.0	5.5	6.9	18.0	11.0	15.4
Mouth, pharyngeal	219	4.3	7.9	8.3	11.1	30.3	17.4	28.2
Esophageal	118	11.5	19.8	21.0	26.1	53.9	38.1	31.6
Laryngeal	23	6.4	24.0	24.0	32.3	61.8	45.6	34.3
Lung	4441	8.7	14.0	15.1	19.1	44.2	29.7	45.5
Stomach	3099	0.3	1.0	1.0	1.5	5.2	2.2	4.8
Liver	2807	1.9	1.0	1.6	1.5	5.6	2.9	4.0
Pancreatic	2152	0.6	1.8	1.8	2.7	9.6	4.8	9.8
Colorectal	3448	1.1	1.6	1.8	2.4	8.4	3.7	4.2
Kidney^b^	247	3.0	5.2	5.6	7.6	23.8	12.8	4.8
Bladder^c^	451	5.9	7.1	8.5	10.8	30.1	14.6	16.4
Cervical	826	7.2	6.2	7.5	7.9	22.4	14.3	3.4
Leukemia	626	0.4	0.7	0.7	1.0	3.8	2.0	1.6
Total CVDs^d^	30 858	4.7	6.4	7.5	9.6	27.7	12.7	7.5
IHD	6854	4.0	9.2	9.7	13.7	37.5	19.5	6.3
Stroke	13 246	1.3	1.1	1.2	1.2	5.3	3.6	2.7
Diabetes	5671	4.3	8.8	9.5	13.3	35.2	18.5	1.8
Respiratory diseases^e^	8664	6.7	12.1	13.6	17.4	37.0	19.9	26.1
COPD	1868	5.3	26.8	27.1	36.9	66.6	41.9	62.9
Asthma	981	19.9	26.8	30.3	36.9	66.2	42.1	.
Lower RTI	5079	4.5	5.5	6.8	8.7	25.0	10.0	.
Pulmonary TB	736	8.1	1.0	4.0	1.7	5.9	2.1	8.5

COPD, Chronic obstructive pulmonary disease; CPS-II, Cancer Prevention Study-II; CVD, cardiovascular disease; IHD, ischemic heart disease; KCPS, Korean Cancer Prevention Study; RTI, respiratory tract infection; SAF, smoking attributable fraction of deaths; TB, tuberculosis.

^a^KCPS, adjusted: SAF estimated using SIR for participants 75–79 and ≥80 years of age based on the estimated lung cancer mortality rates of KCPS smokers according to a log-binomial regression analysis.

^b^CPS-II: kidney and other urinary tract cancer (number of deaths in 2012 = 396).

^c^CPS-II: bladder cancer (number of deaths in 2012 = 302).

^d^IHD: stroke, hypertensive disease, and other CVDs (I10–I99).

^e^COPD: asthma, lower RTI, and pulmonary TB.

KCPS-SIR-based SAFs were as follows: 18.0% for cancers, 27.7% for CVD, and 37% for respiratory diseases. Adjusted KCPS-SIR-based SAFs were 11% for cancers, 12.7% for CVD, and 19.9% for respiratory diseases. CPS-II-SIR-based SAFs were lower than KCPS-SIR-based SAFs, except for lung cancer and COPD, with differences of >20 percentage points for several diseases, including IHD, diabetes, and esophageal cancer. CPS-II-SIR-based SAFs were larger or smaller than adjusted KCPS-SIR-based SAFs depending on the disease assessed.

In females, SIR-based SAFs were significantly larger than prevalence-based SAFs. There was a difference of approximately 17 percentage points between KCPS-SIR- and lag20yr-CS prevalence-based SAFs, which were >20 percentage points for the majority of diseases. Adjusted KCPS-SIR- and lag20yr-CS prevalence-based SAFs differed by <5 percentage points for most diseases.

## DISCUSSION

This study is the first conducted in an Asian population to estimate and compare the SAFs of deaths by the most commonly used methods using the country’s own lung cancer mortality rates of smokers and never-smokers and RRs of various diseases associated with smoking, all of which exhibit differences compared to Western countries. SIR- and prevalence-based SAFs were similar in males, but SIR-derived estimates were significantly higher than prevalence-based SAFs in females. SIR-based SAF estimates differed among reference cohorts according to lung cancer mortality rate, disease RRs, smoking history, and other factors.

The 20-year-lagged prevalence-based SAFs were similar to those in a recent study of SAFs for cancer in Korea in 2009.^33^ However, SAFs in females in that study were slightly lower than values in our study. This might be caused by the application of a lower prevalence of smokers among the elderly and the use of RRs from different data sources. Other studies^24^^,^^34^ reported higher SAFs for CVD deaths (37% and 31% among males) because the higher prevalences of smokers from 1980 or higher RRs (1.7 and 2.2) were applied to older ages.

Among prevalence-based SAFs, differences according to lag-time were marginal in males, whereas SAFs obtained using longer-lagged prevalence periods were significantly larger in females. The prevalence of current smokers among Korean males peaked in 1980 (79.3%) and has continued to decrease during the past 30 years to 43.3% in 2012.^12^^,^^13^^,^^35^ However, because the prevalence of ever-smokers in each 10-year birth cohort was stable, SAF estimates were similar among males when the prevalences and RRs of former smokers were included in analyses. Among Korean females, changes in prevalence of smokers were relatively marginal (12.6% in 1980; 7.7% in 1990; 5%–7.4% thereafter) except in those ≥60 years of age (47.2% in 1980; 2.4% in 2012). Differences according to time-lag period were largely attributable to the cohort of females in their 70s and 80s in 2012; the prevalences of smokers among females in their 70s and 80s in 2012 were approximately 3.7% and 0.9% in the analysis with no lag, approximately 6% and 18% with a 10-year lag, and 11.3% and 29.5% with a 20-year lag. Furthermore, SAF differences according to current prevalence of smokers were not redeemed by former prevalence of smokers and RRs.

In countries exhibiting a decreasing trend in prevalence of smokers, using current prevalence is a conservative method of estimating deaths attributable to smoking.^5^ When using the lag-time approach, risk decline after cessation^7^ should be considered. For lung cancers, >15 years of smoking cessation was required to reduce the lung cancer risk to the level of never-smokers.^7^^,^^20^^,^^36^ Risk decline after cessation is assumed to be similar for other cancers. For IHD and stroke, risk among former smokers decreased more rapidly than risk for lung cancer, becoming equivalent to that of never-smokers after 10–14 years.^25^^,^^36^^–^^38^ Using a 20-year-lagged prevalence method, the inclusion of prevalence and RR of former smokers could result in SAF overestimation. The 10-year-lagged prevalence method may represent the most appropriate choice for evaluating most diseases. In the GBD study, SIR was used to estimate exposure to smoking for cancers and chronic respiratory disease, with 10-year-lagged prevalence used for all other outcomes.^1^

Particularly in females, KCPS-SIR-based SAFs were larger than SAF estimates derived using other methods. This difference was largely due to the high SIR of 1, which was caused by the unexpectedly low lung cancer mortality rates in KCPS smokers ≥75 years of age (Figure FigureB). Such low lung cancer mortality rates in old age were not observed in the general Korean population in 2012 but were consistent with those observed among the Korean population during 1992–1998 (ie, the time period identical to that of the follow-up of KCPS smokers). This is unlikely to be due to the low cumulative hazard of smoking, as the smoking prevalence in the 1980s, when these individuals would have been in their 50s or 60s, was higher than at any other time in the 30 years since 1980.^12^^,^^13^^,^^35^ Low lung cancer mortality rates during 1992–1998 were probably due to under-diagnosed cancer deaths among the elderly,^39^^–^^41^ as suggested by the fact that the proportion of cancer deaths to all deaths among those ≥75 years of age increased by 15 percentage points between 1992 and 2012.^32^ Furthermore, the characteristics of smokers in the general population may increasingly differ from those of the reference cohort’s smokers over time.^6^ If the low lung cancer mortality rates of the reference cohort’s smokers were caused by factors other than smoking, SIR-based SAFs might be overestimated. Therefore, we estimated the lung cancer mortality rates of older age groups using a log-binomial regression model and calculated the adjusted KCPS-SIRs accordingly. However, the suspicion of dissimilarity between smokers represented by KCPS and those in the current general Korean population and the need for the modeled values reduced our preference for KCPS-SIR- and adjusted KCPS-SIR-based SAFs.

Compared with SAFs of other countries, KCPS-SIR-based SAFs for cancer in Korea were similar to those of developed countries and Japan but were lower than those of the United States.^6^^,^^26^^,^^42^ CPS-II-SIR-based SAFs for each type of cancer and COPD were similar to the results from the Institute for Health Metrics and Evaluation (IHME)^43^ of the GBD 2010,^1^ although the re-estimated CPS-II RRs were used. CPS-II-SIR-based SAFs for cancers and COPD and those from the IHME were similar to the SAFs from the IHME in Japan and Singapore and lower than those of developed countries and the United States.^43^ SAFs derived from the GBD 2010 study tended to be lower than SAFs derived from prevalence- and KCPS-SIR-based methods among Korean males. This tendency was similar to those in Japanese studies.^26^^,^^27^^,^^43^^,^^44^

CPS-II-based SAFs were markedly smaller than KCPS-SIR- and prevalence-based SAFs for most diseases, except lung cancer and COPD. The marked difference in SAF estimates by reference population may not be surprising. The background SIR was estimated under the assumption that the RR for lung cancer mortality was constant across countries.^5^^,^^7^ However, the RR of smoking-associated lung cancer mortality in Korea and Japan is significantly lower than in Western countries.^19^ This is partly attributable to the lower level of exposure among smokers, the higher lung cancer mortality rates in nonsmokers caused by a less-strict definition and greater exposure to environmental tobacco smoke, and lifestyle or genetic differences. Regardless of the cause of this discrepancy, the risk excess (that is, RR − 1) for lung cancer mortality in the CPS-II was approximately five times higher than that of the KCPS, resulting in a much lower SIR. This SIR, which represents smoking exposure based on lung cancer risk, is applied to the calculation of the SAFs for other diseases. Consequently, the CPS-II-based SAFs were much smaller for the diseases for which RRs were not as high as the RRs for lung cancer. Furthermore, the smoking history of the study population may be very different from that of the reference population, thus invalidating the assumption underlying SIR that excess risks for lung cancer and other diseases are in a constant ratio among all subpopulations defined by smoking categories.^45^ Therefore, CPS-II-based SAFs may be less suitable for a population with very different RRs for lung cancer and other diseases and smoking history than those derived using other methods.

The difference between SIR- and prevalence-based SAFs among females was greater than that among males. A large discrepancy among females was also found in a Japanese study, and those authors considered the discrepancy to be due to a large difference between smoking exposure measures (ie, SIR and current smoker prevalence).^46^ Oza et al suggested several explanations for the discrepancy in estimates according to study methods.^6^ In our study, the difference between SIR- and prevalence-based SAFs among females might be due to underestimation of smoker prevalence. In a Korean study, cotinine-verified smoking rates were 5.2 percentage points higher than self-reported rates.^47^ The Korean government’s attempts to regulate tobacco use, together with social pressure and a traditional, Confucianism-based culture, could account for the underreporting of female smoking in Korea. When we assumed that prevalence rates of current and former smokers among females in 2001 were both higher by 5 percentage points in all age groups, estimated SAFs accorded more closely with adjusted KCPS-SIR-based SAFs, although they were still lower. In addition, lung cancer mortality rates among female smokers in KCPS might be underestimated due to underreported smoker prevalence (prevalence 4%)^16^^,^^17^ and under-diagnosed lung cancer deaths (99 cases) during a follow-up period of only 6 years,^18^ even though KCPS is a large cohort including 480 000 females.^16^ Another reason may be that the rates of ill-defined causes of deaths were higher among females in the 1990s,^40^^,^^41^ so lung cancer mortality rates may have been underestimated and SIR overestimated in comparison to males. However, such a large difference between the two methods among females could not be sufficiently explained, and the cause of such a difference remains unclear.

This study has several limitations. First, we used the age-specific RRs for CVDs and COPD from a Japanese study due to the lack of data from Korean studies. Second, the 6-year follow-up period for lung cancer mortality rates in the KCPS cohorts seemed to be short for observation of risk of related diseases. Finally, we did not cover the secondhand smoking-attributable burden because the SIR method is not usually used for estimating deaths attributable to secondhand smoking.

Despite such limitations, this study contains important implications for estimating smoking-attributable deaths in countries with mortality rates and disease risks different from Western countries. Currently, either prevalence- or SIR-based methods can be used to estimate SAFs for Korean males. However, prevalence-based methods would be more appropriate than SIR-based methods for females. We cannot be sure that the lung cancer mortality rates of the cohort’s smokers were stable and comparable to those of current smokers. On the other hand, more valid prevalence data may be obtained through the development of a survey system. If more valid exposure data were available, the prevalence-based method would be more appropriate.

In conclusion, prevalence- and SIR-based methods can both be used to estimate smoking-attributable deaths, but both methods have limitations. Therefore, a population’s smoking history, lung cancer mortality rates, disease risks, and tendency toward underreporting of smoking behavior, as well as data availability, should all be considered when selecting a method of estimating smoking-attributable deaths.

## References

[1] Lim SS, Vos T, Flaxman AD, Danaei G, Shibuya K, Adair-Rohani H, A comparative risk assessment of burden of disease and injury attributable to 67 risk factors and risk factor clusters in 21 regions, 1990–2010: a systematic analysis for the Global Burden of Disease Study 2010. Lancet. 2012;380:2224–60. 10.1016/S0140-6736(12)61766-8 23245609 PMC4156511

[2] Husten CG. Tobacco use (ICD-10 F17). In: Remington PL, Brownson RC, Wegner MV, editors. Chronic disease Epidemiology and Control. Washington, DC: American Public Health Association; 2010.

[3] Brønnum-Hansen H, Juel K. Estimating mortality due to cigarette smoking: two methods, same result. Epidemiology. 2000;11:422–6. 10.1097/00001648-200007000-00010 10874549

[4] Pérez-Ríos M, Montes A. Methodologies used to estimate tobacco-attributable mortality: a review. BMC Public Health. 2008;8:22. 10.1186/1471-2458-8-22 18211696 PMC2262075

[5] Tachfouti N, Raherison C, Obtel M, Nejjari C. Mortality attributable to tobacco: review of different methods. Arch Public Health. 2014;72:22. 10.1186/2049-3258-72-22 25126417 PMC4128614

[6] Oza S, Thun MJ, Henley SJ, Lopez AD, Ezzati M. How many deaths are attributable to smoking in the United States? Comparison of methods for estimating smoking-attributable mortality when smoking prevalence changes. Prev Med. 2011;52:428–33. 10.1016/j.ypmed.2011.04.007 21530575

[7] Ezzati M, Lopez AD. Smoking and oral tobacco use. In: Ezzati M, Lopez AD, Rodgers A, Murray CJ, editors. Comparative quantification of health risks: global and regional burden of disease attributable to selected major risk factorsm volume I. Geneva, Switzerland: World Health Organization; 2004.

[8] Ezzati M, Henley SJ, Lopez AD, Thun MJ. Role of smoking in global and regional cancer epidemiology: current patterns and data needs. Int J Cancer. 2005;116:963–71. 10.1002/ijc.21100 15880414

[9] Ezzati M, Henley SJ, Thun MJ, Lopez AD. Role of smoking in global and regional cardiovascular mortality. Circulation. 2005;112:489–97. 10.1161/CIRCULATIONAHA.104.521708 16027251

[10] Ezzati M, Lopez AD. Estimates of global mortality attributable to smoking in 2000. Lancet. 2003;362:847–52. 10.1016/S0140-6736(03)14338-3 13678970

[11] Peto R, Lopez AD, Boreham J, Thun M, Heath C Jr. Mortality from tobacco in developed countries: indirect estimation from national vital statistics. Lancet. 1992;339:1268–78. 10.1016/0140-6736(92)91600-D 1349675

[12] Korea Centers for Disease Control and Prevention. Korea Health Statistics 2012: Korea National Health and Nutrition Examination Survey (KNHANES V-3). Osong Health Technology Administration Complex, Korea: Korea Centers for Disease Control and Prevention; 2013.

[13] Korea Institute for Health and Social Affairs. In-depth analyses of the 2001 National Health and Nutrition Examination Survey: The Health Interview and Health Behavior Survey Part. Seoul, Korea: Korea Institute for Health and Social Affairs; 2003.

[14] Jee SH, Foong AW, Hur NW, Samet JM. Smoking and risk for diabetes incidence and mortality in Korean men and women. Diabetes Care. 2010;33:2567–72. 10.2337/dc10-0261 20823342 PMC2992192

[15] Jee SH, Golub JE, Jo J, Park IS, Ohrr H, Samet JM. Smoking and risk of tuberculosis incidence, mortality, and recurrence in South Korean men and women. Am J Epidemiol. 2009;170:1478–85. 10.1093/aje/kwp308 19917554 PMC2800271

[16] Jee SH, Ohrr H, Sull JW, Yun JE, Ji M, Samet JM. Fasting serum glucose level and cancer risk in Korean men and women. JAMA. 2005;293:194–202. 10.1001/jama.293.2.194 15644546

[17] Jee SH, Samet JM, Ohrr H, Kim JH, Kim IS. Smoking and cancer risk in Korean men and women. Cancer Causes Control. 2004;15:341–8. 10.1023/B:CACO.0000027481.48153.97 15141135

[18] Thun MJ, Hannan LM, Adams-Campbell LL, Boffetta P, Buring JE, Feskanich D, Lung cancer occurrence in never-smokers: an analysis of 13 cohorts and 22 cancer registry studies. PLoS Med. 2008;5:e185. 10.1371/journal.pmed.0050185 18788891 PMC2531137

[19] Marugame T, Sobue T, Satoh H, Komatsu S, Nishino Y, Nakatsuka H, Lung cancer death rates by smoking status: comparison of the Three-Prefecture Cohort study in Japan to the Cancer Prevention Study II in the USA. Cancer Sci. 2005;96:120–6. 10.1111/j.1349-7006.2005.00013.x 15723657 PMC11158599

[20] Wakai K, Marugame T, Kuriyama S, Sobue T, Tamakoshi A, Satoh H, Decrease in risk of lung cancer death in Japanese men after smoking cessation by age at quitting: pooled analysis of three large-scale cohort studies. Cancer Sci. 2007;98:584–9. 10.1111/j.1349-7006.2007.00423.x 17425595 PMC11158971

[21] Thun M, Day-Lally C, Myers D, Calle E, Flanders W, Zhu B, et al. Trends in tobacco smoking and mortality from cigarette use in Cancer Prevention Studies I (1959 through 1965) and II (1982 through 1988). In: National Cancer Institute, Smoking and Tobacco Control Monograph 8: Changes in cigarette-related disease risks and their implications for prevention and control. NIH Publication no. 97-4213. Washington D.C.: NIH publication; 1997. p. 305–82.

[22] Thun MJ, Apicella LF, Henley SJ. Smoking vs other risk factors as the cause of smoking-attributable deaths: confounding in the courtroom. JAMA. 2000;284:706–12. 10.1001/jama.284.6.706 10927778

[23] Kang HY, Kim HJ, Park TK, Jee SH, Nam CM, Park HW. Economic burden of smoking in Korea. Tob Control. 2003;12:37–44. 10.1136/tc.12.1.37 12612360 PMC1759072

[24] Jee SH, Yun JE, Park JY, Sull JW, Kim IS Smoking and cause of death in Korea: 11 years follow-up prospective study. Korean J Epidemiol. 2005;27:182–90 (in Korean).

[25] Honjo K, Iso H, Tsugane S, Tamakoshi A, Satoh H, Tajima K, The effects of smoking and smoking cessation on mortality from cardiovascular disease among Japanese: pooled analysis of three large-scale cohort studies in Japan. Tob Control. 2010;19:50–7. 10.1136/tc.2009.029751 20008160

[26] Ikeda N, Inoue M, Iso H, Ikeda S, Satoh T, Noda M, Adult mortality attributable to preventable risk factors for non-communicable diseases and injuries in Japan: a comparative risk assessment. PLoS Med. 2012;9:e1001160. 22291576 10.1371/journal.pmed.1001160 PMC3265534

[27] Katanoda K, Marugame T, Saika K, Satoh H, Tajima K, Suzuki T, Population attributable fraction of mortality associated with tobacco smoking in Japan: a pooled analysis of three large-scale cohort studies. J Epidemiol. 2008;18:251–64. 10.2188/jea.JE2007429 19075498 PMC4771610

[28] Danaei G, Ding EL, Mozaffarian D, Taylor B, Rehm J, Murray CJ, The preventable causes of death in the United States: comparative risk assessment of dietary, lifestyle, and metabolic risk factors. PLoS Med. 2009;6:e1000058. 10.1371/journal.pmed.1000058 19399161 PMC2667673

[29] Hanley JA. A heuristic approach to the formulas for population attributable fraction. J Epidemiol Community Health. 2001;55:508–14. 10.1136/jech.55.7.508 11413183 PMC1731931

[30] Lilienfeld A, Lilienfeld D. Foundations of Epidemiology. New York: Oxford University Press; 1980.

[31] Levin ML. The occurrence of lung cancer in man. Acta Unio Int Contra Cancrum. 1953;9:531–41. 13124110

[32] Statistics Korea. Cause of Death: Deaths and death rates by cause/by sex/by age (five-year age). Daejeon, Republic of Korea. Statistics Korea; 2012.

[33] Park S, Jee SH, Shin HR, Park EH, Shin A, Jung KW, Attributable fraction of tobacco smoking on cancer using population-based nationwide cancer incidence and mortality data in Korea. BMC Cancer. 2014;14:406. 10.1186/1471-2407-14-406 24902960 PMC4090397

[34] Kim JY, Ko YJ, Rhee CW, Park BJ, Kim DH, Bae JM, Cardiovascular health metrics and all-cause and cardiovascular disease mortality among middle-aged men in Korea: the Seoul male cohort study. J Prev Med Public Health. 2013;46:319–28. 10.3961/jpmph.2013.46.6.319 24349653 PMC3859853

[35] Korea Institute for Health and Social Affairs. In-depth analyses of the Third National Health and Nutrition Examination Survey: The Health Interview and Health Behavior Survey Part. Seoul, Korea: Korea Institute for Health and Social Affairs and Korea Centers for Disease Control and Prevention; 2007.

[36] Kawachi I, Colditz GA, Stampfer MJ, Willett WC, Manson JE, Rosner B, et al. Smoking cessation and decreased risks of total mortality, stroke, and coronary heart disease incidence among women: a prospective cohort study. In: National Cancer Institute, Smoking and Tobacco Control Monograph 8: Changes in cigarette-related disease risks and their implications for prevention and control. Bethesda MD: National Cancer Institute; 1997.

[37] Iso H, Date C, Yamamoto A, Toyoshima H, Watanabe Y, Kikuchi S, Smoking cessation and mortality from cardiovascular disease among Japanese men and women: the JACC Study. Am J Epidemiol. 2005;161:170–9. 10.1093/aje/kwi027 15632267

[38] Kondo T, Osugi S, Shimokata K, Honjo H, Morita Y, Maeda K, Smoking and smoking cessation in relation to all-cause mortality and cardiovascular events in 25 464 healthy male Japanese workers. Circ J. 2011;75:2885–92. 10.1253/circj.CJ-11-0416 21979146

[39] Jo MW, Khang YH, Yun S, Lee JY, Lee MS, Lee SI. Proportion of death certificates issued by physicians and associated factors in Korea, 1990–2002. J Prev Med Public Health. 2004;37:345–52 (in Korean). 25175616

[40] Kim B A development of the cause of death statistics. Journal of The Korean Official Statistics. 1999;4:1–30 (in Korean).

[41] Park S, Lee T Analysis and Improving ways of Factors affecting the Ill-defined Causes of Death of the Aged in Korea. Korean J of Health Policy & Administration. 2011;21:329–48 (in Korean) 10.4332/KJHPA.2011.21.2.329

[42] Peto R, Lopez A, Boreham J, Thun MJ. Mortality from smoking in developed countries 1950–2000 (2nd edition); c2006. Available from: http://www.ctsu.ox.ac.uk/deathsfromsmoking/publications.html.

[43] Institute for Health Metrics and Evaluation (IHME). GBD Database. Seattle, WA: IHME, University of Washington; c2014 [updated March, 2013; Cited April 12, 2015]. Available from: http://vizhub.healthdata.org/gbd-cause-patterns/.

[44] Inoue M, Sawada N, Matsuda T, Iwasaki M, Sasazuki S, Shimazu T, Attributable causes of cancer in Japan in 2005—systematic assessment to estimate current burden of cancer attributable to known preventable risk factors in Japan. Ann Oncol. 2012;23:1362–9. 10.1093/annonc/mdr437 22048150

[45] Lee PN. Mortality from tobacco in developed countries: are indirect estimates reliable? Regul Toxicol Pharmacol. 1996;24:60–8. 10.1006/rtph.1996.0064 8921546

[46] Murakami Y, Miura K, Okamura T, Ueshima H; EPOCH-JAPAN Research Group. Population attributable numbers and fractions of deaths due to smoking: a pooled analysis of 180 000 Japanese. Prev Med. 2011;52:60–5. 10.1016/j.ypmed.2010.11.009 21111753

[47] Jung-Choi KH, Khang YH, Cho HJ. Hidden female smokers in Asia: a comparison of self-reported with cotinine-verified smoking prevalence rates in representative national data from an Asian population. Tob Control. 2012;21:536–42. 10.1136/tobaccocontrol-2011-050012 21972062

